# Effects of COVID-19 Infection Control Measures on Appointment Cancelation in an Italian Outpatient Memory Clinic

**DOI:** 10.3389/fpsyt.2020.599844

**Published:** 2020-11-30

**Authors:** Gianfranco Spalletta, Desirée Estela Porcari, Nerisa Banaj, Valentina Ciullo, Katie Palmer

**Affiliations:** ^1^Laboratory of Neuropsychiatry, Department of Clinical and Behavioral Neurology, IRCCS Santa Lucia Foundation, Rome, Italy; ^2^Menninger Department of Psychiatry and Behavioral Sciences, Baylor College of Medicine, Houston, TX, United States; ^3^Department of Neuroscience, University of Rome Tor Vergata, Rome, Italy; ^4^Department of Internal Medicine and Geriatrics, Università Cattolica del Sacro Cuore, Rome, Italy

**Keywords:** COVID-19, SARS-CoV-2 coronavirus, neurocognitive disorders, non-communicable disease, NCD

## Abstract

**Background:** In the first wave of the COVID-19 pandemic in 2020, many countries made changes to the routine management of patients with non-communicable diseases, including neurocognitive disorders. Therefore, many “so-called” non-urgent elective procedures and outpatient appointments have been canceled or postponed, possibly impacting negatively on health and well-being of patients in the short- and long-term.

**Aim:** Here, we aimed at describing numbers and types of outpatient appointments canceled as a result of government's restrictive measures in our memory clinic.

**Methods:** The scheduled appointments at the memory clinic of the Santa Lucia Foundation IRCCS, Rome, Italy, are recorded in a comprehensive dataset under strict administrative control. Here, we compared appointments (first-time and follow-up) that were canceled from January to April 2020 with those of the corresponding months in 2019.

**Results:** We observed a substantial decrease in appointments during 2020. The majority of scheduled appointments were follow-up, and about a quarter were first-time appointments. We estimated that 66.7% and 77.4% of patients missed out respectively their first and follow-up appointments in our memory clinic due to government's restrictive measures in March–April 2020.

**Conclusions:** A large number of patients with neurocognitive disorders missed crucial appointments due to government's restrictive measures, and many experienced a delay in initial diagnosis and initiation of treatment. This has relevant impact on their treatment and consequently has (is still having and potentially will have) an increase on the healthcare service burden of clinics. Furthermore, as a second wave of COVID-19 affects Europe, and with winter approaching, it is a compelling priority to ensure easy and rapid access to appropriate assessment, care and treatment in the event of a new outbreak and potential subsequent lockdowns, with particular attention to the development of specific healthcare technologies customized to older persons with cognitive impairment.

## Introduction

Since the first case of Severe Acute Respiratory Syndrome—Coronavirus-2 (SARS-Cov-2) was reported and confirmed in Wuhan in December 2019 (1) there have been a series of governments' restrictive measures worldwide to reduce the spread of the pandemic. In Italy, according to article 13 of the law decree number 14 of the march 9th 2020 of the President of the Italian Republic, each region had the possibility to suspend all non-urgent healthcare services. Consequently, elective procedures and appointments were canceled throughout the national territory.

Many countries have employed similar restrictive measures (2–4) and have made changes to the routine management of patients with non-communicable diseases [e.g., canceling “so-called” non-urgent appointments, which have important implications for the identification and treatment, and therefore for the progression of these chronic conditions (5)]. Although the focus on procedures to urgently slow SARS-CoV-2 infection rates and minimize the number of infected individuals has extreme importance, it has short- and long-term negative consequences on health and well-being of patients with non-communicable diseases, including neurocognitive disorders (NCD) (6–10).

In particular, this may affect not only the diagnosis of new-onset mild and major NCD but also have a potential negative effect on neuropsychiatric symptoms, medication adherence, and disease progression (11). In fact, many surveys have been structured to measure these aspects, specifically in patients during the appointment suspension and for future quarantines (12, 13). Until the pandemic is under regulation control, we will be unable to establish the extent that the postponement of routine clinical care has on persons with mild and major NCD.

In Italy, different types of health and socio-health services are available for people with NCD. According to the Observatory of Dementias from the Italian National Institute of Health there are 591 memory clinic in the whole country (data from the 2015 census) and more than three million people are directly involved in the formal and informal care of individuals with Alzheimer's disease nationwide (14). It is, therefore, of utmost importance to assess the impact of the pandemic-related changes to routine clinical management of persons with NCD.

In the present study we focused on patients with NCD whose neuropsychiatric care changed due to the first COVID-19 outbreak. Specifically, we aimed at comparing the appointments that were performed during the lockdown period (January to April 2020) to the same period of the previous year. In addition, based on the requests' reason, we classified the appointments into “first-time” and “follow-up” to determine how many people would experience a delay in receiving a new diagnosis, because of first-time appointment missed, and to estimate how many routine clinical follow-up care has been disrupted.

## Materials and Methods

The memory clinic of the Santa Lucia Foundation IRCCS is an outpatient clinic where patients with mild and major NCD are referred. Typically, after a first-time appointment, which includes psychiatric, neurological and neuropsychological assessment, patients attend follow-up appointments in which they are generally diagnosed using blood sampling, neuroimaging and other procedures and, if appropriate, prescribed therapy that is confirmed or gradually adjusted. Each appointment is recorded in a comprehensive dataset under strict administrative control.

In the present study, we verified the number of patients with scheduled appointments at the memory clinic at the Santa Lucia Foundation IRCCS from January to April 2020. We then recorded the number of appointments that were canceled due to the government-enforced reduction of non-urgent healthcare services in Italy during the lockdown and compared these numbers with the appointments in the corresponding months of the previous year (January-April 2019). Records were independently checked by two neuropsychologists to determine whether the examination was (i) a follow-up appointment in patients already attending the memory clinic, (ii) a first-time appointment, and (iii) the reasons for requesting a first appointment.

## Statistical Analysis

We compared the number of daily appointments conducted in the same period in 2019 and 2020 (January—April) using a *z*-test (α = 0.05) to determine statistically significant differences between proportions.

## Results

**Figure 1:**
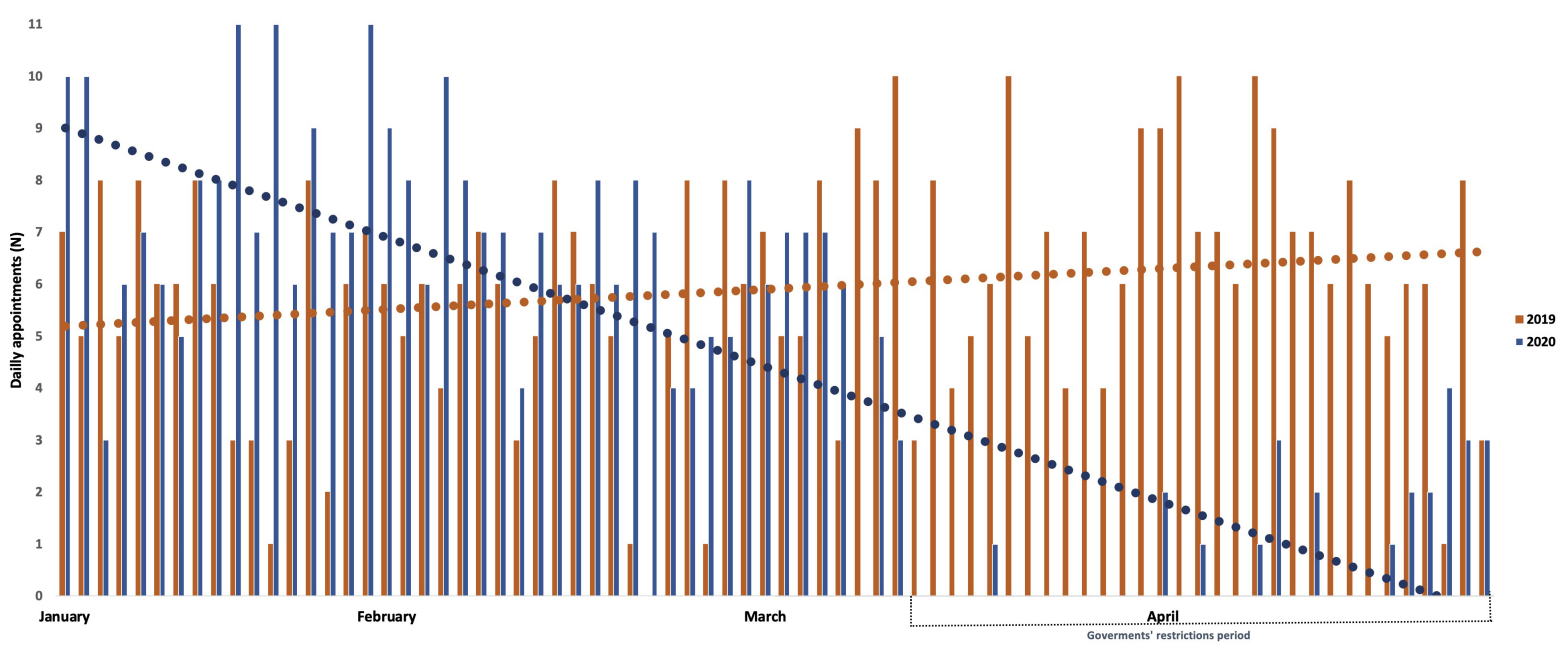
Number of daily appointments conducted during January–April 2019 and 2020.

**Figure 2:**
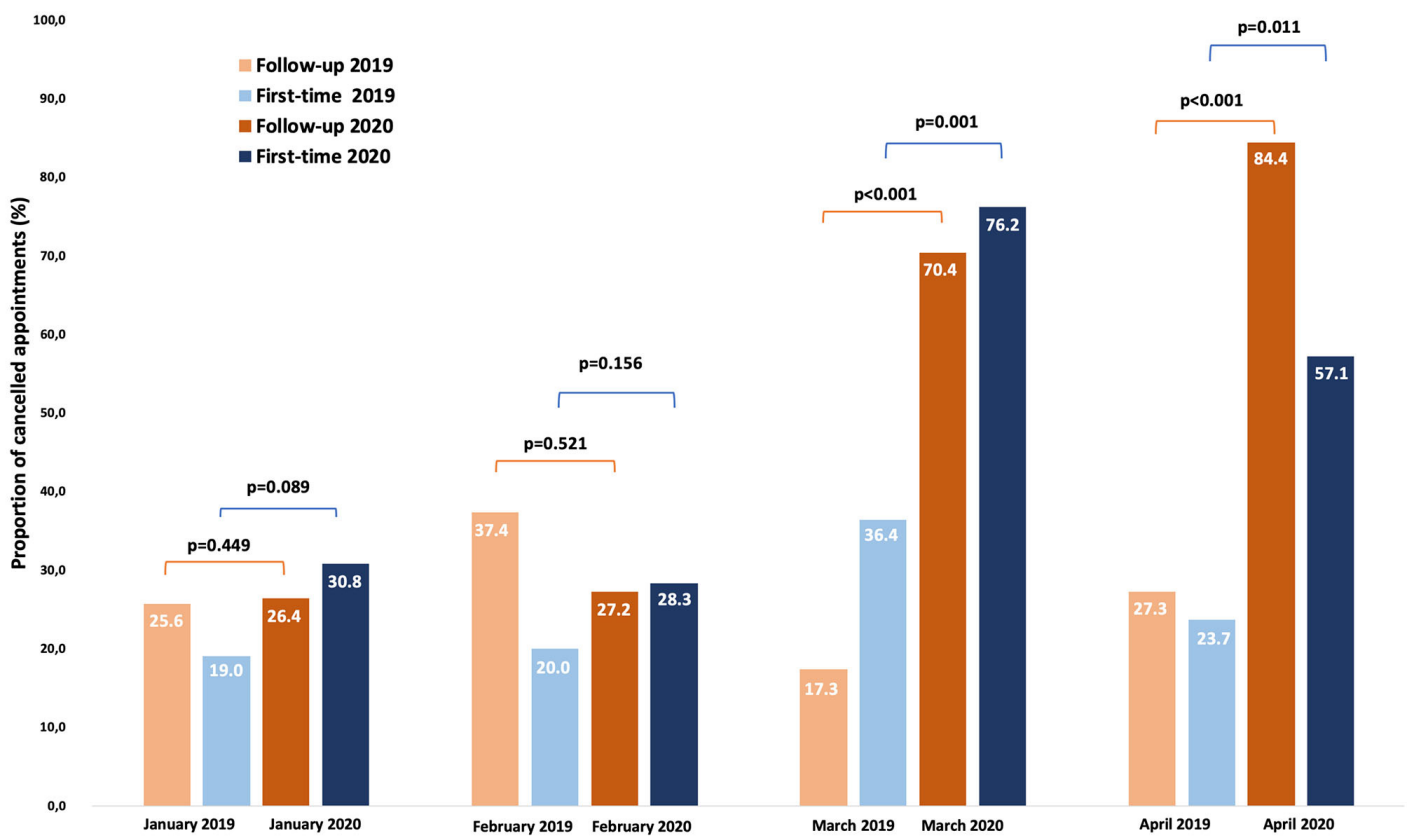
Proportion of canceled appointments in 2019 compared to 2020.

Figure 1 shows the number of daily appointments conducted in the same months of 2019 and 2020 (January to April). We quantified a considerable decrease in appointments specifically during March-April 2020 and the vast majority of scheduled appointments were follow-up (Table 1). In such period, a total of 251 scheduled appointments were canceled (follow-up *N* = 211; first-time *N* = 40). Figure 2 reports the proportion of canceled appointments (first-time and follow-up) in 2019 compared to 2020. Specifically, March and April show an increase in canceled appointments in 2020 compared to the previous year with 70.4% and 84.4% of follow-up appointments respectively, while only 17.3% and 27.3% were canceled in the same periods in 2019. Further, 76.2% (March) and 57.1% (April) of first-time appointments were canceled in 2020 respect to the 36.4% and 23.7% in the same months in 2019. In general, 72% of patients missed out their appointments during the lockdown period (i.e. 66.7% first-time and 77.4% of follow-up). There was a significant difference in the proportion of canceled follow-up (*p* < 0.001) and first-time (*p* = 0.001) appointments respectively in March and April 2020 compared to the same periods in 2019. As shown in Table 2 (Reasons for scheduling a first-time appointment at the memory clinic), most patients (85.2%) were referred to our clinic for the first-time for a new diagnostic evaluation, while the remaining were either patients enrolled in clinical trials or those who already had a dementia diagnosis but require the adjustment or initiation of pharmacological treatment. Almost half of the first-time appointments were for people with cognitive disturbance that requires evaluation.

**Table 1:** Number of appointments scheduled and conducted during 2019 and 2020.

	**Total**	**Follow-up**		**First-time**	
	***n***	***n***	***(%)***	***n***	***(%)***
**Scheduled appointments**					
**2019**					
January	120	78	65.0	42	35.0
February	149	99	66.4	50	33.6
March	171	127	74.3	44	25.7
April	159	121	76.1	38	23.9
**2020**					
January	190	125	65.8	65	34.2
February	185	125	67.6	60	32.4
March	150	108	72.0	42	28.0
April	174	160	92.0	14	8.0
**Conducted appointments**					
**2019**					
January	92	58	63.0	34	37.0
February	102	62	60.8	40	39.2
March	133	105	78.9	28	21.1
April	121	88	72.7	29	24.0
**2020**					
January	137	92	67.2	45	32.8
February	134	91	67.9	43	32.1
March	42	32	76.2	10	23.8
April	31	25	80.6	6	19.4

**Table 2:** Reasons for scheduling a first-time appointment at the memory clinic.

**Reason**	***n***	***%***
Diagnostic exam: Referrals for cognitive disturbances (memory, language, attention, orientation)	82	48.2
Diagnostic exam: Screening for cognitive functioning due to a familial case of a memory disorder	33	19.4
Diagnostic exam: Patient referrals for examination by another regional physician	30	17.6
Persons with an existing dementia diagnosis requiring evaluation/adjustment of pharmacological treatment	21	12.4
Patients enrolled in a clinical trial requiring the adjustment of pharmacological treatment or initiation of standard therapy	4	2.4

## Discussion

The COVID-19 outbreak dramatically hit Italy at the end of January 2020 and the government's restrictive measures included in the decree number 14 resulted in the cancelation of the majority of appointments in memory clinics. Our data highlight that many patients with pre-existing NCD missed potentially important follow-up appointments, and that a substantial number of first-time visit NCD had a delay in diagnoses. Considering the high prevalence of behavioral disorders in patients with NCD (e.g., depression, apathy, psychomotor, and psychotic syndromes), that 60% of NCD patients reported a rapid increase during quarantine (12) and that they are associated with acceleration of cognitive decline (15), such a delay in diagnosis and treatment may have serious consequences on patient outcomes. Moreover, these estimates suggest that the reduction in services will result in a large influx of first-time appointments (previously scheduled ones plus new first-time appointments) with a consequent lengthening of waiting lists and increased burden on already stretched healthcare services.

It is possibly premature to estimate the long-term effects that patients will experience as a result of missing their routine appointments. We, however, are confident that a number of patients will experience a worsening of symptoms due to lack of pharmacological control or support from psychologists and psychiatrists (16, 17). Moreover, the social restrictions and increased isolation may lead to increased negative effects on memory disorders and neuropsychiatric symptoms of older people with NCD (18, 19). In fact, recent data on this topic reported an increase of behavioral symptoms in elderly with dementia (8–10). It is also possible that there may be an increased need for assessment of new patients in geriatric psychiatry settings as a result of the negative consequence of quarantine and social distancing measures. In fact, psychological symptoms due to stressor events can contribute to cognitive decline (20) and social isolation, reduced social network, and loneliness can lead to generalized anxiety and major depression disorders in older individuals (21–23). Further, during lockdown it is likely that many older persons reduced their physical activity levels (5), which may impair symptoms; for example, cardiorespiratory fitness is also associated with cognitive functioning in older persons (24). This scenario may exacerbate psychological distress in caregivers, condition which may further worsen patients' behavioral symptoms, acting in a vicious loop of mutual increase of psychiatric burden (9).

NCD is a major public health concern and timely diagnosis is important for improving the course of illness and initiating appropriate therapeutics and care planning. Our study provides some absolute numbers in terms of how many patients who needed a first-time neuropsychiatric diagnostic evaluation in our clinic missed out on personalized care during the COVID-19 pandemic. In particular, a total of 72% of patients that scheduled an appointment during March and April 2020 missed it, with approximately a quarter of these being new patients, most of whom were referred for diagnostic evaluation. Diagnoses are made as a result of a process which usually takes time, in which the patient undergoes a series of procedures in order to gradually identify underlying pathologies. This means that, as a parallel effect, there will be a delay in diagnosing new cases of NCD, which may affect treatment and progression of the disorders as well as access to health and social care services (25).

In the current public health emergency, Italy was generally unprepared for digital healthcare approaches for managing patients, although some other countries had better training and facilities to keep in touch with patients remotely (26–28). Across the EU Member States there are still two fifths (40%) of older people (aged 65–74 years) who have never used a computer and this number is higher than two thirds in Italy (29). The low percentage of older people who are able to use technology (e.g., personal computer, internet, and other devices) is a barrier in terms of digitalization of medicine in Italy. However, the other obstacle is that only a small percentage of Italian medical services are equipped and trained to use healthcare technologies. So even if technology use increases in older persons, the healthcare system may not be able to provide digital medical support in a large number of cases (30).

Several pilot projects have focused on the usability of different types of technologies for older people with mild and major NCD in test laboratories or at home (e.g., PETAL and ReMember-Me projects, within Active & Assisted Living Programme - Horizon 2020); what emerged is a benefit for both patients and their caregivers, especially in terms of quality of life, occupational performance, and human dignity (31–33). In general, the use of Information and Communications Technology (ICT) by people with NCD is well-tolerated if devices are specifically designed to be easy to use for the target users and if they receive adequate training in order to learn how to use the technology and avoid issues due to their cognitive problems (34). Thus, one priority area for preparing Italy for potential future outbreaks of COVID-19 is to design and develop telemedicine solutions for individuals with cognitive impairment [i.e., remote memory clinics (35)], and to prepare adequate training for both healthcare professionals and patients.

Some limitations of the study should be discussed. The data only provide information about one memory clinic. It would be interesting to compare present data with those of others memory clinics in Italy or Europe. However, all Italian outpatient clinics adhered to the same government guidelines during the pandemic, which suggests that our results may be generalizable to those of the others. However, there may be differences in other countries depending on their infection-control strategy, especially in less developed countries. Therefore, our findings may not be fully applicable to low- or middle-income countries. Research in the short-term should focus on what effect the appointment cancelations had on patients in terms of their disease progression, cognitive functioning, and behavioral symptoms. Currently, healthcare professionals in the Italian National Health System are urgently trying to ease the backlog of patients by rescheduling canceled appointments. These efforts are still ongoing and continued infection-control limitations mean that many patients still have not returned for a clinical examination. Further, it will be interesting to compare disease and symptom progression in patients from countries that employed different policies to cope with the pandemic. Future research also needs to urgently develop and assess digital and telehealth alternatives with the aim to avoid the adverse consequence of a new period of social lockdown. In particular, it is important to find long-term alternatives to face-to-face outpatient services, potentially ICT and telehealth solutions that can support both patients and caregivers remotely (36–42). These solutions need to be user-friendly for people with cognitive and sensory impairment, for example including intuitive interface, clear and understandable symbols, big fonts, fewer commands and with colors that could help patients to remember different functions (43, 44). In general, telemedicine can reduce the risk for the development of negative outcomes in mental health precipitated by the reduction of social contact and less access to health services, improving dementia symptoms management like psychological and behavioral symptoms (45).

## Conclusion

As expected, the data from our memory clinic in Rome showed that a large number of patients with NCD missed potentially important follow-up appointments during the pandemic, and many will experience a delay in initial diagnosis and beginning of treatment. Since COVID-19 continues to make its presence felt in healthcare all over the world, it is a compelling priority to ensure an easy and rapid access to appropriate assessment, care, and treatment in the event of a new outbreak and potential lockdown. Hence, a great challenge now is to convert this global emergency into a source of change that will see an implementation of telemedicine use by the healthcare system, alongside to traditional face-to-face medicine, developing specific technologies customized to older persons with cognitive impairment.

## Data Availability Statement

The raw data supporting the conclusions of this article will be made available by the authors, without undue reservation.

## Author Contributions

GS and KP conceived the structure and content of the paper. DEP, NB, and KP wrote the paper. NB and VC provided revisions of the paper. All authors contributed to the article and approved the submitted version.

## Conflict of Interest

The authors declare that the research was conducted in the absence of any commercial or financial relationships that could be construed as a potential conflict of interest.
